# Vitrification of Human Germinal Vesicle Oocytes: before or after *In Vitro* Maturation?

**DOI:** 10.22074/ijfs.2017.4717

**Published:** 2017-02-16

**Authors:** Evangelia Kasapi, Byron Asimakopoulos, Katerina Chatzimeletiou, Stamatios Petousis, Yannis Panagiotidis, Nikos Prapas, Nikos Nikolettos

**Affiliations:** 1 Iakentro Fertility Centre, IVF Laboratory, Thessaloniki, Greece; 2 Democritus University of Thrace, Laboratory of Physiology, Faculty of Medicine, School of Health Sciences, Alexandroupolis, Greece; 3 Aristotle University of Thessaloniki, Medical School, 1st Department of Obstetrics and Gynaecology, Papageorgiou General Hospital, Thessaloniki, Greece; 4 Embryokosmogenesis, IVF Laboratory, Alexandroupolis, Greece

**Keywords:** Vitrification, *In Vitro* Maturation, Meiotic Spindle, Fertility Preservation

## Abstract

**Background:**

The use of immature oocytes derived from stimulated cycles could be of great importance, particularly for urgent fertility preservation cases. The current study aimed to determine whether *in vitro* maturation (IVM) was more successful before or after vitrification of these oocytes.

**Materials and Methods:**

This prospective study was performed in a private *in vitro* fertilization (IVF) center. We collected 318 germinal vesicle (GV) oocytes from 104 stimulated oocyte donation cycles. Oocytes were divided into two groups according to whether vitrification was applied at the GV stage (group 1) or *in vitro* matured to the metaphase II (MII) stage and then vitrified (group 2). In the control group (group 3), oocytes were *in vitro* matured without vitrification. In all three groups, we assessed survival rate after warming, maturation rate, and MII-spindle/chromosome configurations. The chi-square test was used to compare rates between the three groups. Statistical significance was defined at P<0.05 and we used Bonferroni criterion to assess statistical significance regarding the various pairs of groups. The Statistical Package for the Social Sciences version 17.0 was used to perform statistical analysis.

**Results:**

There was no significant difference in the survival rate after vitrification and warming of GV (93.5%) and MII oocytes (90.8%). A significantly higher maturation rate occurred when IVM was performed before vitrification (82.9%) compared to after vitrification (51%). There was no significant difference in the incidence of normal spindle/ chromosome configurations among warmed oocytes matured *in vitro* before (50.0%) or after (41.2%) vitrification. However, a higher incidence of normal spindle/chromosome configurations existed in the *in vitro* matured oocytes which were not subjected to vitrification (fresh oocytes, 77.9%).

**Conclusion:**

In stimulated cycles, vitrification of *in vitro* matured MII oocytes rather than GV oocytes seems to be more efficient. This approach needs to be verified in nonstimulated fertility preservation cases.

## Introduction

The advent of vitrification in clinical practice has opened new possibilities and options for oocyte cryopreservation. Currently, oocyte vitrification is widely used in most *in vitro* fertilization (IVF) laboratories as a routine procedure (1-3). The high survival rates that follow warming have made oocyte banking feasible for heterologous use, fertility preservation for social reasons, in cases where women face the dangers of premature ovarian failure, as well as prior to chemoor radiotherapeutic treatments (4-6). Vitrification of oocytes may easily serve as a rescue plan in IVF cycles when the male partner is not able to produce a semen sample at the time of oocyte retrieval. Under the above circumstances, a number of immature oocytes are often retrieved from a stimulated cycle. The majority of collected oocytes from *in vitro* maturation (IVM) cycles are immature and at the germinal vesicle (GV) stage (7). GV oocytes collected for fertility preservation in women who need an urgent onset of chemo- or radiotherapeutic treatments may be of great value since they can be either matured and cryopreserved or fertilized and cultured *in vitro* (8, 9). In such cases, the question is whether oocytes should be vitrified before or after IVM in order to maintain the highest developmental competence (10, 11).

Published data show conflicting results, mostly due to the multiple steps of this procedure and different outcomes of each step. Egerszegi et al. (12) have applied vitrification on GV or *in vitro* matured metaphase II (MII) pig oocytes. They reported a higher survival rate after vitrification-warming for MII compared to GV oocytes. However, both groups had similar maturation rates; most importantly, the GV vitrified group had better spindle configuration and F-actin integrity. The study concluded that vitrification at the GV stage was more advantageous in terms of cleavage and blastocyst formation compared to the *in vitro* matured MII oocytes. On the other hand, many studies that compared vitrification of immature GV oocytes and *in vitro* matured MII oocytes concluded that poor maturation and low fertilization rates were major problems associated with the vitrification of GV oocytes (13-18). It is widely accepted that oocytes which fail to mature *in vivo* under ovarian gonadotropin stimulation and human chorionic gonadotrophin (hCG) trigger are intrinsically abnormal. They present a high incidence of aneuploidies and low developmental competence compared to oocytes that have reached the MII stage at the moment of retrieval (19-22). Additionally, the probability of denuded GV oocytes to mature *in vitro* after vitrification and warming is significantly reduced due to the loss of oocytecumulus cell communication (23).

In the present study, the recruitment of immature oocytes from stimulated cycles of oocyte donors represented an experimental model that used easily available material and aimed towards the configuration of a methodology to rescue immature oocytes of significant value in natural or IVM cycles. We sought to investigate whether IVM might be more successful before or after vitrification. GV stage oocytes derived from stimulated cycles were subjected to IVM before or after vitrification/warming and monitored in terms of survival rate, maturation rate, and the status of spindle/chromosome configuration. The control group consisted of non-vitrified GV oocytes matured *in vitro*.

## Materials and Methods

We conducted this prospective study from January 2013 until September 2014 in a private assisted reproduction unit. The Institutional Review Board (ref. no. 9/2012, 14 November, 2012) approved this study and we obtained informed consents from all couples that received eggs from their dedicated donors. The IVF Unit computerized database contains all patient characteristics as well as parameters related with stimulation protocol and gamete handling. These data are regularly recorded and revalidated on a monthly basis by specialized personnel in order to maintain data reliability. GV oocytes were obtained from controlled ovarian hyperstimulation oocyte donation cycles. Oocyte donors had a mean age of 26 ± 2 years. We randomly allocated the GV oocytes into three groups. Group 1 (GV vitrification) immature oocytes were first vitrified, subsequently warmed and matured *in vitro*. Group 2 (MII vitrification) immature oocytes underwent IVM and those that reached the MII stage were subsequently vitrified and warmed. Group 3 (no vitrification) GV oocytes were placed in maturation medium and served as the control group. We assessed MII spindle configuration by immunostaining in all three groups. MII oocytes that presented with partial or complete disorganization of the spindle poles or complete absence of the meiotic spindle were characterized as abnormal.

### Ovarian stimulation and oocyte retrieval

We used a fixed 6-day gonadotropin-releasing hormon (GnRH)-antagonist protocol (Orgalutran 0.25 mg, Organon) with 225 IU/day of recombinant follicle stimulation hormone (FSH, Puregon, Organon) that started on day 2 of the cycle for ovarian stimulation (19). The daily dose of recombinant FSH was adjusted according to the donor’s ovarian response based on serum estradiol concentrations and the number and size of ovarian follicles. hCG (10000 IU, Pregnyl, Organon) was administered when three or more follicles >17 mm in mean diameter were present on ultrasound and a serum estradiol concentration of >1500 pg/ml. Donors at risk of hyperstimulation received an additional dose of GnRH-antagonist on the day before hCG administration (24). The retrieved oocytes were incubated for 2 hours in equilibrated Quinn’s Advantage Fertilization (HTF, Sage, Copper Surgical, USA) supplemented with 5% human serum albumin (HSA, Sage, Copper Surgical, USA) at 37ºC and 6% CO_2_. Afterwards, they were denuded by using 80 IU/ml hyaluronidase solution (Sage, Copper Surgical, USA).

### In vitro maturation

We placed the GV oocytes in maturation medium (Sage, Copper Surgical, USA) supplemented with 75 mIU/ml FSH, 75 mIU/ml luteinizing hormone (LH), and 10% serum substitute supplement (SSS, Sage, Copper Surgical, USA) for 24-48 hours after oocyte denudation. Next, we determined oocyte maturation by the presence of the first polar body. The total time of incubation after oocyte retrieval was 26 hours until the first control and 50 hours until the second control, or 62 and 86 hours post hCG administration, respectively.

### Oocyte cryopreservation

We used a closed carrier system (VitriSafe, VitriMed, Austria) for oocyte vitrification (25). All chemical substances were purchased by Sigma-Aldrich unless otherwise mentioned. A mixture of dimethyl sulphoxide (DMSO) and ethylene glycol (EG) was used in dilutions of 1.25%/1.25%, 2.5%/2.5%, 5%/5% and 10%/10% for the respective equilibration steps (ES1 to ES4) and 20%/20% for the vitrification step (VS), supplemented with 100 mg of Ficoll and 0.5 mol/l sucrose. For all solutions, the basal medium consisted of Quinn’s Advantage Medium w/HEPES (Sage, Copper Surgical, USA) supplemented with 20% of SSS (Sage, Copper Surgical, USA). Briefly, the oocytes were placed for 3 minutes in each 50 μl ES1, ES2, and ES3 microdrop and for 6 minutes in a microdrop of ES4. Subsequently, oocytes were placed in a VS drop (100 μl) for approximately 60 seconds, which included the time needed to load the oocytes on the carrier, enclose them in a protective straw, and seal and plunge the oocytes into liquid nitrogen. During the cryopreservation steps, all solutions were maintained at room temperature (22ºC).

### Oocyte warming

We warmed the oocytes by serially placing them into sequential step down sucrose solutions (1 mol/l, 0.75 mol/l, 0.5 mol/l, 0.25 mol/l, and 0.125 mol/l) in Quinn’s Advantage Medium w/HEPES (Sage, Copper Surgical, USA) supplemented with 20% SSS (Sage, Copper Surgical, USA). Briefly, we cut the upper part of the protective straw and removed VitriSafe using an extractor tool. The tip of the carrier was immediately immersed in 1 ml of the 1 mol/l sucrose solution at 37ºC. After 1 minute, we placed the oocytes in 0.75 mol/l sucrose for an additional 1 minute, followed by 2 minutes in 0.5 mol/l, 2 minutes in 0.25 mol/l, and 1 minute in 0.125 mol/l sucrose. These steps were performed at room temperature. The oocytes that survived after warming with no signs of cytoplasmic degeneration or zona damage were cultured either in IVM medium (GV oocytes) or in Quinn’s Advantage Fertilization (HTF) for MII oocytes (Sage, Copper Surgical, USA).

### Spindle chromosome configuration analysis

Oocyte immunostaining was performed according to Chatzimeletiou et al. (26, 27) using a primary rat monoclonal antibody specific for α-tubulin (AbD Serotec, Oxford, UK) to visualize microtubules in combination with 4,6-diamidino-2-phenylindole (DAPI) to visualize DNA. Briefly, all oocytes were rapidly fixed in ice cold methanol, washed in Ca++/Mg++-free phosphate-buffered saline (PBS, Gibco BRL) that contained 2% bovine serum albumin (BSA), transferred into 10 ml drops of the primary antibody under mineral oil, and incubated at 4ºC for 1 hour. The oocytes were subsequently washed in PBS/BSA and transferred to 10 μl drops of the secondary antibody, which was highly cross-adsorbed Alexa Fluor 488 or 594 conjugates (Invitrogen, USA) that contained 1 ng/ml DAPI. After a one-hour incubation in the secondary antibody, the oocytes were washed in PBS/BSA, mounted on slides in Vectashield antifade medium (Vector Laboratories, CA, USA) under a coverslip, and examined using a fluorescence microscope (Zeiss Axioskop) and/or laser scanning confocal microscope (Leica TCS-SP). We classified spindle abnormalities according to criteria previously described by Chatzimeletiou et al. (28). A spindle with barrel shaped poles and with chromosomes aligned at the equator was classified as normal. A spindle with one or two poorly defined or apparently absent poles, generally with misaligned chromosomes was classified as having an abnormal shape. Spindles with more than two poles were classified as multipolar. Finally, any chromosomes not aligned with the other chromosomes on the spindle were classified as lagging chromosomes which might occasionally result in chromosome loss.

### Primary and secondary outcomes

Primary outcomes included overall maturation rate, normal spindle configuration rate, and survival rate after warming. The maturation rate 62 hours after hCG administration was considered a secondary outcome. We defined the survival rate as the ratio of oocytes that survived after warming to the overall number of vitrified oocytes. The overall maturation rate was defined as the ratio of the number of final MII oocytes to the number of oocytes that underwent IVM. The maturation rate after 62 hours was defined as the ratio of MII 62 hours after hCG to the overall number of oocytes that underwent IVM. Finally, the normal spindle configuration rate was defined as the ratio of oocytes that had a normal spindle after IVM to the total number of oocytes that survived in each group.

### Statistical analysis

The categorical variables were expressed as percentages. We used the chi-square test to compare rates between the three groups. Statistical significance was defined at P<0.05 while Bonferroni criterion was used to assess statistical significance regarding the various group pairs (group 1 versus group 2, group 1 versus group 3, group 2 versus group 3). Odds ratios (OR) with 95% confidence intervals (CI) were estimated for comparisons between pairs of groups.

## Results

In this study, there were 318 oocytes (107 in group 1, 105 in group 2, and 106 in group 3). We compared the survival rate after warming between groups 1 and 2 as no vitrification was included in the protocol for group 3 oocytes. There was no significant difference between the two groups. The survival rate was 93.5% for group 1 (100/107 cases) and 90.8% for group 2 (79/87 cases, P=0.487, Table 1). Maturation rate significantly differed in groups 2 and 3 versus group 1. The overall maturation rate was 82.9% for group 2 (87/105) versus 51% for group 1 (51/100, OR: 4.6, 95% CI: 2.4-8.8, P<0.001). Similarly, the overall maturation rate significantly differed between groups 3 (81.1%) and 1 (51%, P<0.001). No significant difference existed between groups 2 and 3 (P=0.736, Table 1).

The maturation rate 62 hours after hCG significantly differed (P<0.001) among the study groups with 27.0% for group 1 (n=27), 60.0% for group 2 (n=63), and 67% for group 3 (n=71). Group 2 had a 4-fold increase compared to group 1 (ΟR: 4.06, 95% CI: 2.25-7.31, P<0.001), whereas group 3 had a 5-fold increase compared to group 1 (ΟR: 5.49, 95% CI: 3.01-9.98, P<0.001). There was no significant difference observed between groups 2 and 3 (P=0.529). Group 3 had a significantly different normal spindle configuration rate of 77.9% (67/86 cases) compared to 41.2% for group 1 (21/51 cases, P<0.001) and 50.0% for group 2 (35/79 cases, P<0.001). However, no significant difference existed between vitrification groups 1 (41.2%) and 2 (50.0%, P=0.797, Figes.1, 2).

**Table 1:** Main outcomes in the study groups


	Group 1 n=107	Group 2 n=105	Group 3 n=106	P value

Survival rate	93.5% (100/107)	90.8% (79/87)		0.487
Overall maturation rate	51% (51/100)	82.9% (87/105)	81.1% (86/106)	<0.001^*^
Maturation rate 62 hours after hCG	27% (27/100)	60% (63/105)	67% (71/106)	<0.001^*^
Normal spindle configuration	41.2% (21/51)	50.0% (35/79)	77.9% (67/86)	<0.001^#^


*; Statistical significance of the differences between group 1 versus group 2 and group 1 versus group 3 and #; Statistical significance of the difference between group 3 versus group 1 and group 3 versus group 2.

**Fig.1:**
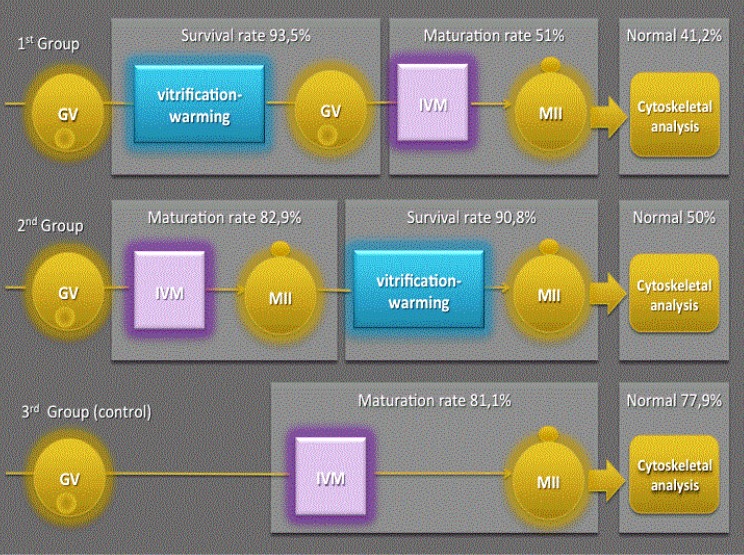
Graphical illustration of primary outcomes for the three study groups. GV; Germinal vesicle, IVM; *In vitro* maturation, and MII; Metaphase II.

**Fig.2:**
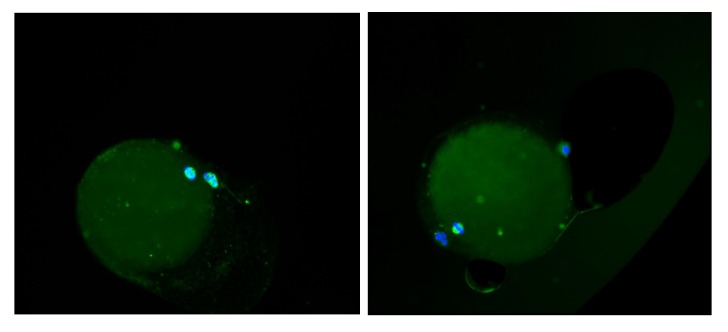
Spindle chromosome configuration in oocytes after *in vitro* maturation (IVM). A. Germinal vesicle (GV) oocyte cryopreserved and subsequently matured *in vitro* to metaphase II (MII) following warming. Note the normal spindle formation and B. GV oocyte cryopreserved and warmed following IVM. Note the normal spindle formation [α-tubulin stained is green and DNA labeling with 4,6-diamidino-2-phenylindole (DAPI) is blue].

## Discussion

Results from previous studies indicate that vitrification of GV oocytes from IVM cycles is a feasible option (29-31). Various proposed protocols aim to optimize vitrification to keep synchronized the processess of nuclear and cytoplasmic oocyte maturation and maintain further, normal embryo development (32, 33). The immature oocytes assessed in the present study have been retrieved from stimulated cycles and were, by definition, of low developmental potential (34). However, they could still be used as a research model to investigate the influence of vitrification on immature human oocytes.

Our results, being in accordance with recent published data (10, 35), showed no significant difference in the survival rate of immature oocytes between vitrification at the GV (93.5%) and the MII (90.8%) stages, even with a closed vitrification protocol. However, the maturation process appeared to be compromised when intercepted by the vitrification/warming step. GV oocytes vitrified before IVM had a significantly lower maturation rate (51%) compared to oocytes that were *in vitro* matured before vitrification of note, approximately half of the GV oocytes that matured after vitrification needed a longer culture time in maturation media in order to reach the MII stage compared to those that underwent IVM before vitrification. These findings agreed with results from a similar study by Wang et al. (36). Data from both studies did not verify the hypothesis and general belief that the most appropriate time to vitrify oocytes would be at the GV rather than MII stage (12, 37). Although, at the GV stage, the chromatin is diffused in the diplotene state of prophase I and well protected by the nuclear membrane, which suggests that these oocytes are less vulnerable to the risk of chromosome missegregations. This study has demonstrated that GV vitrification significantly compromised the progress of oocyte maturation and rate of fertilization (6, 36).

The cooling and warming procedure seems to affect spindle/chromosome configurations regardless of the developmental stage of the vitrified oocytes (38-40). The cytoskeleton is quite sensitive to environmental changes, such as temperature, during *in vitro* manipulations (40). In the present study, abnormal spindle configurations have been equally found in oocytes from vitrification groups 1 and 2. Both groups presented a higher rate of abnormal configurations compared to the control group with no vitrification.

On the other hand, Hosseini et al. (41) reported that nucleo-cytoplasmic interactions which supported early embryonic development could be damaged during vitrification. Cytoplasmic, rather than nuclear insufficiencies, were generally the major cause of low developmental competence of embryos derived from vitrified oocytes. Lei et al. (42) provided evidence that the low developmental competence of vitrified *in vitro* matured oocytes might be attributed to a mitochondrial membrane dysfunction. The authors reported no significant change at the meiotic spindle configuration after vitrification, which conflicted with results of the present study and those reported by Chian et al. (43) who observed that vitrification was related with abnormal spindle and chromosome configurations in the warmed oocytes. Huang et al. (35) compared *in vitro* and *in vivo* matured mouse oocytes. They stated that the poor development of embryos after combined IVM and vitrification might be related to the rate of DNA fragmentation and toxicity of the cryoprotectants, but not to the chromosomal abnormalities. These researchers observed no increase in aneuploidies in the vitrification group. However, Vanderzwalmen et al. (44) reported that the intracellular concentration of cryoprotectants (and toxicity) during vitrification was much lower than the cryoprotectant concentration present in the vitrification solution and the intracellular concentration during slow freezing. Chatzimeletiou et al. (28) demonstrated that following vitrification, spindle abnormalities in human embryos increased. These abnormalities were mainly due to dehydration and mechanical stress sustained by the cells and not cryoprotectant toxicity. However, DNA repair mechanisms might be activated to rescue both oocytes and embryos following vitrification (45).

Despite the fact that embryos derived from *in vitro* matured, vitrified oocytes might be of lower developmental competence, they possibly provide a person’s unique opportunity to fecundity. This attribute has tremendously increased their value and the need to be processed in the best possible way. However, due to the lack of experience on utilization of embryos produced from such oocytes, extensive counseling and prenatal genetic testing should be considered.

## Conclusion

The results of the present study indicated that in terms of survival rate, vitrification of either *in vitro* matured or GV oocytes was equally successful. However, the maturation process of the GV oocytes after vitrification and warming appeared to be compromised. Oocyte vitrification for fertility preservation would likely be more efficient if applied on *in vitro* matured MII oocytes. The present experimental data should be verified by further studies, preferably on non-stimulated cycles where most oocytes are collected at the GV stage.
